# Molecular regulatory mechanism of TRPV4-mediated calcium-dependent 5-HT secretion from enterochromaffin cells and its involvement in ulcerative colitis

**DOI:** 10.1371/journal.pone.0355169

**Published:** 2026-08-13

**Authors:** Shuo Tong, Yujuan Wang, Jianxin Wang, Chengxin Qu, Shude Pang, Wenwen Zhao, Hui Dong

**Affiliations:** 1 Dpartment of Pharmacology, School of Pharmacy, Qingdao University Medical College, Qingdao, China; 2 Department of Pediatric Intensive Care Unit, Shandong Provincial Hospital Affiliated to Shandong First Medical University, Jinan, Shandong, P.R China; Longgang Otorhinolaryngology Hospital & Shenzhen Key Laboratory of Otorhinolaryngology, Shenzhen Institute of Otorhinolaryngology, CHINA

## Abstract

Although acetylcholine (ACh) released by the vagus nerve can stimulate enterochromaffin cells (ECs) to secrete 5-hydroxytryptamine (5-HT), the precise regulatory mechanism underlying this process and its association with the pathogenesis of ulcerative colitis (UC) remain unclear. In this study, we aimed to investigate whether TRPV4-mediated calcium influx in ECs is involved the process of ACh promoting 5-HT secretion which leads to UC. In vitro experiments, western blotting, cellular calcium imaging, and patch clamp were applied to ECs, and dextran sulfate sodium (DSS)-induced UC of mouse model was used in vivo. Carbachol (CCh), a stable analog of ACh, activated mAChR/IP_3_/IP_3_R pathway to increase intracellular [Ca^2+^]_i_levels in ECs, in which both TRPV4 channels and RyR were activated in response to CCh. The selective TRPV4 antagonist HC067047 inhibited the calcium signals and membrane currents induced by IP3/IP3R, as well as 5-HT secretion. In addition, selective blocker of RyR (Dantrolene) also significantly inhibited CCh-induced calcium signals and membrane currents. In vivo experiments, HC067047 significantly suppressed the synthesis and release of 5-HT from ECs and alleviated DSS-induced UC in mice. Taken together, this study reveals a novel mAChR/IP_3_/IP_3_R /TRPV4/5-HT pathway in ECs is likely involved in the pathogenesis of UC. Targeting this pathway holds a promise for the potential prevention and treatment of UC.

## 1 Introduction

Enteroendocrine cells (EECs) are a specialized type of cells in the intestinal epithelium [1]. Although EECs account for only 1% of intestinal epithelial cells, they can secrete at least 25 hormones to regulate metabolism and function of gut and brain in human body, and therefore work as a key component of the gut-brain axis [2,3]. EECs can be subdivided into multiple subtypes, including enterochromaffin cells (ECs), L cells, MX cells, and D cells. Among them, ECs mainly produce and release 5-hydroxytryptamine (5-HT). which is an important intestinal signaling molecule, ECs synthesize and secrete approximately 90%−95% of 5-HT in the human body [4]. However, so far it has not been fully understood about the detailed regulatory mechanisms of 5-HT release from ECs by neurotransmitters in the gastrointestinal (GI) tract.

Acetylcholine (ACh) an important neurotransmitter released from the vagus, not only exerts a crucial role in regulating GI physiological functions but also deteriorating intestinal diseases by regulating hormone secretion and signal transduction [5]. Although previous study indicated that ACh may involve in intestinal diseases by participating in 5-HT secretion [6,7], the specific regulatory mechanisms remain unclear and need further investigation.

It is well-known that the vagus releases ACh to regulate 5-HT secretion from ECs, in which calcium signaling is an essential. Although the vagus released ACh may activate two types of ACh receptors to trigger Ca^2+^ signaling: muscarinic acetylcholine receptors (mAChRs) and nicotinic acetylcholine receptors (nAChRs) [8–10]. It is largely unclear the underlying molecular mechanisms how ACh regulates 5-HT secretion from ECs.

Ulcerative colitis (UC), a major form of inflammatory bowel disease (IBD), exhibits uniqueness in terms of clinical features, pathological mechanisms, and complications [11,12]. In recent years, the incidence of UC has been rising sharply in developing countries and seriously affecting patients’ quality of life [13]. However, the complex disease etiology hinders UC’ prevention and treatment. Therefore, in-depth study of the pathogenesis of UC is an urgent priority.

Clinical study has found that in the patients with UC, serum concentration of 5-HT is significantly increased [14–17], and excessive 5-HT exerts abdominal pain and diarrhea to further aggravate intestinal diseases [18,19]. Moreover, it was previously reported that nAChRs have anti-infammatory effect in GI tract while mAChRs may lead to diarrhea [20,21]. Considering that ACh regulates 5-HT secretion from ECs, mAChR-5-HT may be involved in the pathological process of UC.

The inositol trisphosphate IP_3_/IP_3_R pathway upon acting on cholinergic receptors is one pivotal component of the calcium signaling toolbox [22]. ACh/IP_3_/IP_3_R is the classic signal amplification pathway to mediates [Ca^2+^]_i_ release from the endoplasmic reticulum (ER) [23,24], controlling cytoplasmic and organellar. At present, its downstream pathways are not been elucidated. After [Ca^2+^]_i_ are released from intracellular calcium stores, a variety of calcium-sensitive channels are activated [25]. Among, TRPV4 has particularly high permeability to Ca^2+^ and initiated a series of physiological or pathological processes that are dependent on Ca^2+^ [26,27]. The level of endogenous TRPV4 activators is elevated in patients with UC. Moreover TRPV4 channel blockers effectively alleviate visceral pain symptoms in UC patients [28,29]. Noticeably, in EECs, the release of intracellular calcium through the IP_3_/IP_3_R pathway can further activate TRPV4 channels to induce extracellular calcium influx. It remains unclear whether TRPV4 participates in acetylcholine-regulated 5-HT secretion from ECs. Understanding the ACh-mediated regulation of 5-HT secretion is critical for deciphering its gut physiology and pathology, and may offer therapeutic leads for UC. Thus, we aimed to address two questions: 1) the molecular mechanisms underlying ACh-stimulated 5-HT secretion from ECs; 2) whether ACh-triggered, calcium-dependent 5-HT secretion participates in the pathogenesis of UC. In this study, QGP-1 (a human ECs cell model) was used as a stable and reliable model for native ECs [30,31], because QGP-1 cells are commonly used as a classical 5-HT-secreting cells associated with elevated intracellular Ca^2+^ signaling [32].

## 2 Materials and methods

### 2.1 Cell culture

The small intestinal enteroendocrine cell line QGP-1 was obtained from IMMOCELL (Catalog No. IM-H570, Xiamen, China). The culture medium was composed of RPMI 1640 medium (Catalog No. C11875500BT, Gibco, Shanghai, China) supplemented with 10% fetal bovine serum (FBS, Catalog No. FSP 500, ExCell, Shanghai, China) and 1 vol‰ penicillin/streptomycin (Cat. No. C0222, Beyotime Biotechnology, China). Cells were incubated at 37°C in a humidified atmosphere containing 5% CO_2_, and the medium was changed twice a week. Prior to calcium ion (Ca^2+^) measurement and electrophysiological recording, cells were seeded onto 9-mm coverslips one night in advance to reACh the appropriate density for experiments.

### 2.2 Animal studies and ethics

The study protocol was approved by the Animal Ethics Committee of Qingdao University (Approval No.: QDU-AEC-2025102). All animal care and experimental procedures were performed in accordance with the National Institutes of Health (NIH, USA) Guide for the Care and Use of Laboratory Animals. This study was reported in accordance with the ARRIVE guidelines [33]. Male Kunming mice (6–8 weeks old, weighing 20–22 g) were purchased from Jinan Pengyue Laboratory Animal Co., Ltd. The mice were housed in a controlled environment with constant temperature (25 ± 2 °C) and relative humidity (50 ± 5%), under a 12-hour light/dark cycle, with free access to food and water. After completion of the acetylcholine (eACh) experiment, the mice were placed in a transparent airtight induction chamber with a net volume of 10 L. Pure carbon dioxide (CO_2_) gas was first introduced into the chamber at a flow rate of 3.0 L/min (approximately 30% of the chamber volume per minute) controlled by a precision flowmeter. The mice were continuously exposed to CO_2_ for at least 5 minutes starting from the gas introduction. After confirming the complete cessation of the animals’ breathing via close observation, CO_2_ flow was maintained for an additional 1 minute to ensure the efficacy of euthanasia. Subsequently, the animals were removed from the chamber, and cervical dislocation was performed for secondary confirmation of death. This anesthesia protocol was applied to all animal experiments reported in this study. All animals were randomly assigned to different experimental groups. Data collection and evaluation in all experiments were conducted in a blinded manner, where the experimenters were unaware of the group allocations.

### 2.3 Electrophysiological recordings

In this experiment, patch-clamp recordings were performed using an EPC-10 patch-clamp amplifier and PatchMaster software (HEKA Electronik GmbH) [34]. Patch pipettes were pulled from borosilicate glass (Cat. No. BF150N86, Biospikes, Co. China) using a horizontal micropipette puller (Cat. No. P97, Sutter Instruments, Co. USA), with resistance of 3–5 MΩ. QGP-1 cells on coverslips were fixed in a chamber containing extracellular solution, which consisted of (in mM): 140 NaCl, 5 KCl, 2 CaCl_2_, 2 MgCl_2_, and 10 HEPES, and the pH was adjusted to 7.4 with NaOH. The internal pipette solution was composed of (in mM): 140 CsCl, 0.5 EGTA, 3 Mg-ATP, and 10 HEPES, and the pH was adjusted to 7.2 with NaOH. Recordings were digitized (10 kHz) and stored on a computer, then analyzed using PatchMaster software. Cells were clamped at a voltage of 0 mV to inactivate voltage-gated sodium channels, and 100 ms linear ramps from −100 mV to +100 mV were applied every 2 seconds. Pipette voltages (Vp) are referred to the bath. In the whole-cell configuration, Vp corresponds to the membrane potential, and upward deflections of current traces indicate outward membrane currents. We determined the corresponding current density by calculating the ratio of the measured current to the capacitance value recorded by the amplifier on the membrane. Results were compared with control studies measured on the same day to minimize the effect of day-to-day variability and reported as current results.

### 2.4 Ca^2+^ imaging

Cells were cultured on 9-mm coverslips for 24 hours, then loaded with 5 μM Fura-2 (Cat. No. F8460, Solarbio, China) in an isotonic extracellular buffer containing (in mM): 140 NaCl, 5 KCl, 2 CaCl_2_, 2 MgCl_2_, 10 Glucose, 10 HEPES (pH 7.4), supplemented with 0.01% pluronic F127 (Cat. No. P6791, Solarbio, China) for 60 minutes at 37 °C. The coverslips were placed in a perfusion chamber on the stage of an inverted fluorescence microscope (Olympus IX73, 20 × , Japan). Changes in intracellular Ca^2+^ signaling were measured at excitation wavelengths of 340 nm (for calcium-bound Fura-2) and 380 nm (for calcium-free Fura-2), and an emission wavelength of 510 nm. Experiments were performed at room temperature using MetaFluor software (Molecular Devices, USA). Images were acquired using a digital camera (Cat. No. C11440-42U30, HAMAMATSU, Japan). All Ca^2+^ imaging data were normalized to make the results and conclusions of related experiments clearer.

### 2.5 RNA extraction, reverse transcriptase reaction and RT-qPCR

Intestinal tissues were crushed, and total RNA was isolated using the RNA easy™ Animal Long RNA Isolation Kit (Catalog No. R0027, Beyotime Biotechnology, China) according to the manufacturer’s instructions. RNA concentration was quantified using a Nano-300 Micro-Spectrophotometer (Cat. No. Nano-300, ALLSHENG, China). 1 μg of total RNA was reverse-transcribed at 37 °C for 45 minutes in the presence of 2μl dNTP Mix (10 mM each) (Cat. No. D7168L, Beyotime Biotechnology, China) and 1μl BeyoRT™ II M-MuLV Reverse Transcriptase (Cat. No. D7168L, Beyotime Biotechnology, China). The real-time quantitative PCR protocol was as follows: initial temperature at 95 °C for 30 seconds, followed by 40 cycles of denaturation at 95 °C for 10 seconds and annealing/extension at 60 °C for 30 seconds; the melting curve was analyzed from 65 °C to 95 °C with a temperature increment of 0.5 °C every 5 seconds. PCR amplification was performed using Sybr Green PCR Master Mix (Cat. No. 11202ES08, Yeasen, China) and specific primers for GAPDH and TPH1 (Qingdao Ruibio Biotech Co., Ltd., China). The mRNA expression level of the target gene was normalized using mGAPDH as the reference gene. RT-qPCR was repeated three times. Results were calculated using the 2^-xxct^ method [35].Primers used for RT-qPCR analysis are designed using the online primer-blast tool [36] and listed below.

mGAPDH:
forward 5’-AGGTCGGTGTGAACGGATTTG-3’
backward 5’-GGGGTCGTTGATGGCAACA-3’
TPH1
forward 5’-CAGAGCCAGATACCTGCCATGAAC-3‘
backward 5’- GCCAAGAGAAGCCAAGCCAATTTC-3’
NAChR
forward 5’- GGCAGGATTACCGACTCAACTACAG-3‘
backward 5’- GCAGCCACACGAGTICIGAAGG-3’
M3AChR
forward 5’- CACCCACTTTCCCTTTGATGAACAG-3‘
backward 5’- GGTCTGGCTGGTCGCTTTCC-3’
M1AChR
forward 5’- CAGTGCTACATCCAGTTCCTCTCC-3‘
backward 5’- CGTGCTCGGTTCTCTGTCTCC-3’

### 2.6 ELISA

The ELISA kit for 5-HT was purchased from Enzyme-linked Biotechnology (Cat. No. ml057425 & ml001891, Shanghai Enzyme-linked Biotechnology Co., China). The extraction of cell supernatants and ELISA assay were performed according to the manufacturer’s instructions. Briefly, cells were seeded into 24-well cell culture plates at a density of 2 × 10^5^ cells per well. On the following day, drugs were administered for 2 hours, and then 200 μl of the supernatant was centrifuged at 1 × 10^3^ g for 10 minutes to remove particulate matter. Subsequently, 50 μl of the resulting solution was added to a 96-well plate pre-coated with 5-HT antibody. Various reagents were added in the sequence specified in the instructions. The optical density (OD) of each well was measured at a wavelength of 450 nm. The 5-HT concentration of each sample could be determined from these OD values using a standard curve. For the serum ELISA assay in animals, mice were anesthetized and then euthanized by cervical dislocation. The eyeballs of the mice were enucleated to collect blood. After collection, the blood was allowed to stand at room temperature for 1 hour, then centrifuged at 2500 rpm at 4 °C for 10 minutes. The supernatant of the centrifuged homogenate was retained, and the precipitate below was discarded. The 5-HT content in the serum was detected following the same procedure as described above.

### 2.7 Western blot analysis

Cells were lysed using Western and IP cell lysis buffer (Cat. No. P0013, Beyotime Biotechnology, China), and the supernatant was collected after centrifugation. Protein concentration was determined using a BCA kit (Cat. No. P0010, Beyotime Biotechnology, China). Total cell lysates were separated by SDS-PAGE on a 10% denaturing gel and then transferred to a PVDF membrane (Cat. No. ISEQ00010, Millipore, Billerica, MA, USA). The blot was blocked with 5% non-fat milk at room temperature for 2 hours, then incubated overnight at 4 °C with the following specific primary antibodies: anti-TRPV4 (Cat. No. ACC-034, Alomone, Israel, 1:100) and anti-GAPDH (Cat. No. AG0191, Beyotime Biotechnology, China, 1:1000). After washing, the blot was incubated with horseradish peroxidase (HRP)-conjugated anti-rabbit or anti-mouse secondary antibodies (Cat. No. A0239 and A0216, Beyotime Biotechnology, China, 1:5000) at room temperature for 1 hour. Immunoreactive bands were detected using enhanced chemiluminescence (Cat. No. 34094, Thermo, Waltham, MA, USA).

### 2.8 Immunofluorescence

Tissues were obtained from KM mice. Immediately after euthanasia, colonic tissues were harvested and fixed in 4% paraformaldehyde (PFA) for 24 hours. Subsequently, the tissues were dehydrated in a graded ethanol series, cleared in xylene, and embedded in paraffin. Serial sections (4 μm thick) were cut using a microtome and mounted on poly-L-lysine-coated glass slides, followed by baking in a 60°C oven for 2 hours for subsequent use. Paraffin sections were deparaffinized in xylene I and II for 10 minutes each, then rehydrated in a graded ethanol series (100%, 95%, 85%, 75%) for 5 minutes each, and finally rinsed 3 times with phosphate-buffered saline (PBS, pH 7.4) for 5 minutes per rinse. The sections were placed in preheated sodium citrate buffer (pH 6.0), heated to boiling with high power in a microwave oven, and then maintained at medium-low power for 15 minutes. After cooling naturally to room temperature, the sections were rinsed with PBS.5% bovine serum albumin (BSA) was added dropwise, and the sections were blocked at room temperature for 1 hour to reduce non-specific binding. The blocking solution was discarded, and primary antibody working solution prepared with antibody diluent (Rabbit anti-TPH1, Cat. No.DF6465, Affinity Biosciences, China, 1:200) was added dropwise directly. The sections were placed in a humidified chamber and incubated overnight at 4°C. On the following day, the primary antibody was recovered, and the sections were rinsed with PBS. Corresponding fluorochrome-conjugated secondary antibody (Goat Anti-Rabbit IgG (H + L) Alexa Fluor 488 conjugate, Cat. No. A-11008, Invitrogen, China,1:500) was added dropwise, and the sections were incubated at room temperature for 1 hour in the dark. After rinsing with PBS in the dark, DAPI working solution (1 μg·mL^-1^) was added dropwise for nuclear staining in the dark for 5 minutes. The sections were then rinsed with PBS, mounted with anti-fluorescence quenching mounting medium, covered with coverslips (avoiding air bubbles), and allowed to solidify overnight at room temperature in the dark.

### 2.9 Establishment of animal models of UC

Dextran sulfate sodium (DSS) is commonly used to induce colitis in model mice [37]. After 1 week of acclimation, the mice were randomly divided into three groups: control group (Ctrl group, n = 5), DSS group (2.5% DSS added to drinking water, n = 5), and DSS + HC067047 group (colitis induced by 2.5% DSS for 7 days + intraperitoneal injection of HC067047 at 10 mg·kg^-1^ for 5 days, n = 5). After the administration period, mice were euthanized following the animal euthanasia protocol described in Section 2.2, and colon tissues were collected for H&E staining and colon length measurement. The serum 5-HT level was also detected.

### 2.10 Data and statistical analysis

All quantitative, normally distributed data were analyzed for statistical significance using a Student’s t-test, One-way ANOVA, with Tukey’s post hoc tests when comparing more than two groups relative to a single factor. GraphPad Prism Software (GraphPad Prism version 8.0.2 for Windows; GraphPad Software, www.graphpad.com) was used. (*) indicates P < 0.05, (**) indicates P < 0.01, (***) indicates P < 0.001, (****) indicates P < 0.0001, and (ns) indicates no statistical significance.

## 3 Results

### 3.1 CCh promoted 5-HT secration, elicited intracellular calcium signals and non-selective transmembrane currents in QGP-1 cells

**Fig 1:**
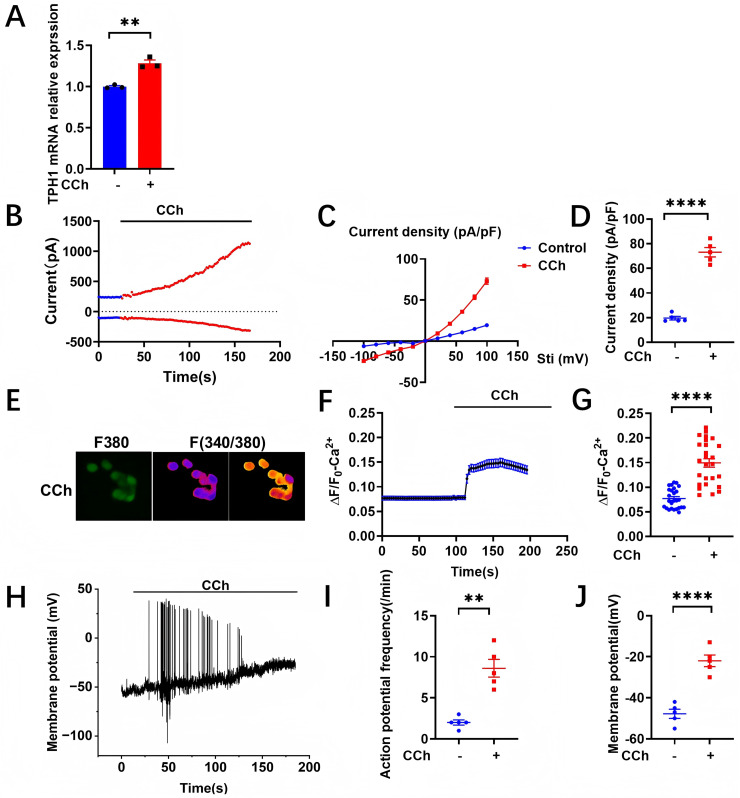
CCh promoted 5-HT secration, elicited intracellular calcium signals and non-selective transmembrane currents in QGP-1 Cells. (A)Intracellular TPH1 transcription data of the control group and the CCh (20 μM)-treated group. (B) CCh (20 μM) increased the transmembrane current of QGP-1 cells at −100 mV or 100 mV. (C)Current density-voltage relationship curves in response to voltage steps from −100 to +100 mV in the presence of CCh (20μM) or Control. (n = 5). (D)Summary data of current density measured at 100 mV. (n = 5). (E)It shows that after the addition of CCh, the fluorescence ratio changes significantly, indicating an increase in intracellular Ca^2+^ concentration. (F) Summary of time-course data of CCh (20 μM)-induced intracellular Ca^2+^ signaling in physiological saline solution (PSS) (n = 26). (G) Comparison of intracellular calcium signals between the control group and the CCh group. (H)Time-voltage graph showing that CCh significantly increases the membrane potential and action potential frequency of QGP-1 cells at a clamped current of 0 pA. (I) Comparison graph of CCh-induced action potential frequency versus the Control group at a clamped current of 0 pA. (J) Comparison graph of CCh-induced membrane potential change amplitude versus the Control group at a clamped current of 0 pA. The statistical significance of differences in the means of experimental groups was determined using Student’s t test or one-way ANOVA followed Tukey by post hoc test for multiple pairwise comparisons. Data were shown as means ± SEM, **P < 0.01, ****P < 0.0001.

It has been documented that under physiological conditions, ACh promotes the excessive release of 5-HT in ECs [6]. The aim of our study to explore related regulation mechanisms in ECs. Due to the instability of ACh, its stable analog CCh was applied in our experiments. The date from qPCR showed that after treating QGP-1 cells with CCh (20 μM) in cell culture medium for 4 hours, the transcriptional level (Fig 1A) of 5-HT synthetase (tryptophan hydroxylase 1, TPH1) in QGP-1 cells was significantly increased. These results indicate that CCh could be the substitute for ACh to avoid its instability. In the following research, patch-clamp technique was utilized to detect the effect of CCh on the membrane current in QGP-1 cells. Transmembrane currents were recorded at 100 mV and −100 mV. After adding CCh (20 μM), a significant increase in membrane current was observed (Fig 1B), indicating that ECs were activated. Meanwhile the current density-voltage relationship curve showed that the involved ion channels were non-selective cation channels when the reversal potential was close to 0 mV (Fig 1C). Statistic analysis from multiple cells further confirmed the significant enhancement of current density induced by CCh (Fig 1D). To verify whether Ca^2+^ could been induced entry into ECs through non-selective cation channels, real-time Ca^2+^ signaling was measured in single QGP-1 cells using the Fura-2 calcium fluorescent dye. As shown in (Fig 1E, 1F), stimulation with CCh (20 μM) indeed triggered significant Ca^2+^ signaling in QGP-1 cells. Statistical analysis was performed on the observed significant enhancement of intracellular Ca^2+^ signaling induced by CCh across multiple QGP-1 cells (Fig 1G). These data indicate that CCh activated a significant enhancement of intracellular Ca^2+^ signaling through non-selective cation channels in QGP-1 cells.

In many excitable cells, activatied calcium signal is one important internal driving force for generating frequency-dependent action potentials, and firing frequency which directly regulate the amount of hormone release [38]. In following experiment, accompanied by activated intracellular Ca^2+^ signaling, membrane potential and action potential frequency of QGP-1 were deteced. The data from electrophysiological patch-clamp technique showed that the changes in membrane potential and action potential over time were induced by CCh (20 μM) under the condition of a clamped current of 0 pA (Fig 1H). The analysis of statistical data from multiple cells (Fig 1I, 1J) further confirm that CCh inducesds an increase in membrane potential and an acceleration in action potential frequency of QGP-1.

Above data demonstrateds that CCh could promote 5-HT secretion by regulating the transduction of intracellular calcium signals.

### 3.2 CCh enhanced intracellular Ca^2+^ signals via mAChR/IP_3_/IP_3_R pathway and activated non-selective cation currents in QGP-1 cells

**Fig 2:**
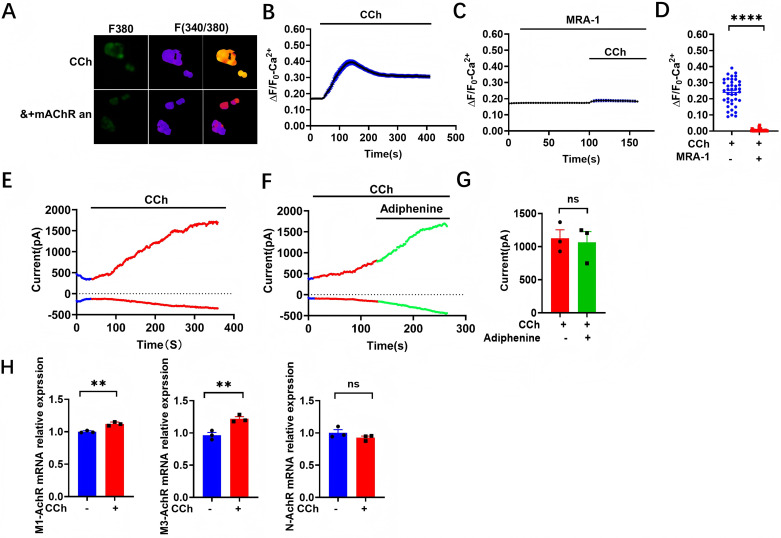
CCh mediates the enhancement of intracellular calcium signaling and the increase of transmembrane current in QGP-1 cells by acting on M-AChR. (A) Shows the changes in fluorescence ratio induced by CCh (20 μM) alone and CCh (20 μM) + MRA-1 (25 μM). (B) Summary of time-course data for calcium signal transduction induced by CCh (20 μM) in PSS solution (n = 42). (C) Summary data of the time course of calcium signals induced by CCh (20 μM) in PSS solution containing MRA-1 (25 μM) (n = 48). (D) Comparison of calcium signals between CCh and CCh + MRA-1 groups. (E) CCh (20 μM) increases the transmembrane current of QGP-1 cells at −100 mV or 100 mV. (F) The enhancement of transmembrane current by CCh (20 μM) cannot be inhibited by Adiphenine (20 μM). (G) Summary data of the maximum transmembrane current in the CCh group and CCh + Adiphenine group (n = 3). (H) Summary of transcription data of M1-AChR, M3-AChR, and N-AChR in QGP-1 cells after treatment with CCh (20 μM). The statistical significance of differences in the means of experimental groups was determined using Student’s t test or one-way ANOVA followed Tukey by post hoc test for multiple pairwise comparisons. Data were shown as means ± SEM, **P < 0.01, ****P < 0.0001.

As a vital cholinergic agonist, CCh could bind to ACh receptors including M and N receptor to activate downstream signaling pathways. In this study, we aimed to identify which receptor subtype was activated by CCh to regulate 5-HT release in QGP-1 cells. First, selective muscarinic cholinergic receptor antagonist (mAChR antagonist 1) and nicotinic cholinergic receptor antagonist were added separately to QGP‑1 cells. As shown in Fig 2A–2D, mAChR antagonist 1 (MRA-1, 25 μM) significantly inhibited the CCh (20 μM)-induced enhancement of intracellular calcium signals, whereas the nicotinic cholinergic receptor (nAChR) antagonist (adiphenine, 20 μM) failed to reverse the CCh(20 μM)-elicited increase in transmembrane current (Fig 2E–2G). Furthermore, results from Fig 2H demonstrated that the transcriptional levels of M1 and M3 receptors were significantly upregulated in QGP‑1 cells following CCh(20 μM) stimulation. Collectively, these findings further confirm that CCh mediates the activation of intracellular calcium signals and the increase in membrane conductance through mAChRs.

As a typical G protein-coupled receptor, activated mAChR leads to a significant increase of intracellular inositol 1,4,5-trisphosphate (IP_3_) levels. Subsequently, IP_3_ acts on IP_3_R, thereby inducing [Ca^2+^]_i_ release from the ER and regulating non-selective cation channels in ECs [39,40]. We aim to further investigate the role of IP_3_/IP_3_R in response to mAChR in QGP-1 cells.

**Fig 3:**
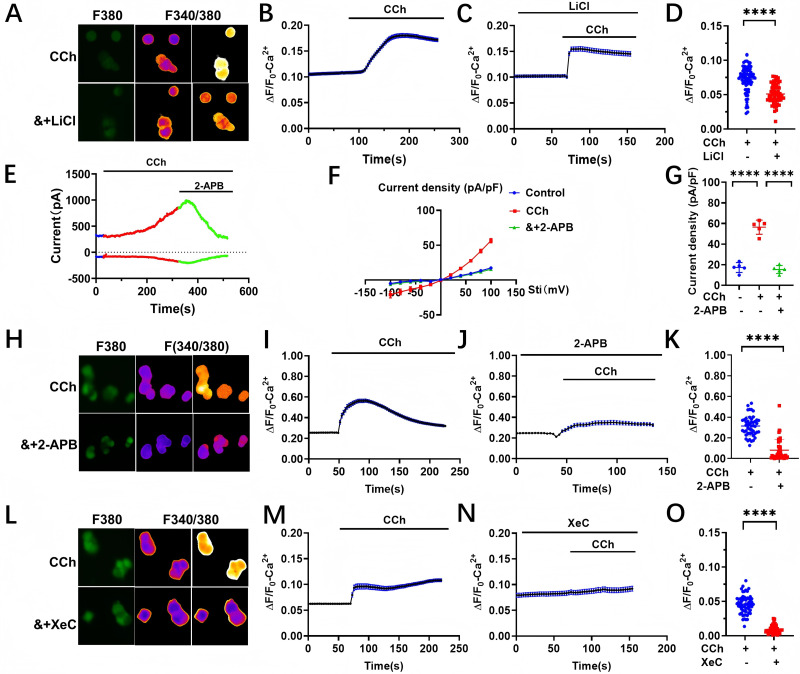
CCh induces the increase of calcium signals and transmembrane current in QGP-1 cells through the IP3 R pathway. (A) Shows the changes in fluorescence ratio induced by CCh (20 μM) alone and CCh(20 μM) + LiCl (1 mM). (B)Summary of time-course data for calcium signal transduction induced by CCh (20 μM) in PSS solution (n = 73). (C) Summary data of the time course of calcium signals induced by CCh (20 μM) in PSS solution containing LiCl (1 mM) (n = 67). (D) Comparison of calcium signals between CCh and CCh + LiCl groups. (E) CCh (20 μM) enhances transmembrane current, and this effect is inhibited by 2-APB (100 μM). (F) Current density-voltage curves in response to voltage steps from −100 to +100 mV in the presence of CCh (20μM) or a combination of CCh plus 2-APB (100μM). (n = 5). (G)Summary data of current density measured at 100 mV. (n = 5). (H)Shows the changes in fluorescence ratio induced by CCh (20 μM) alone and CCh (20 μM) + 2-APB (100 μM). (I) Summary of time-course data for calcium signal transduction induced by CCh (20 μM) in PSS solution (n = 49). (J) Summary data of the time course of calcium signals induced by CCh (20 μM) in PSS solution containing 100 μM 2-APB (n = 50). (K) Comparison of calcium signals between CCh and CCh + 2-APB groups. (L) Shows the changes in fluorescence ratio induced by CCh (20 μM) alone and CCh(20 μM) + XeC (100 nM). (M) Summary of time-course data for calcium signal transduction induced by CCh (20 μM) in PSS solution (n = 50). (N) Summary data of the time course of calcium signals induced by CCh (20 μM) in PSS solution containing XeC (100 nM) (n = 41). (O) Comparison of calcium signals between CCh and CCh + XeC groups. The statistical significance of differences in the means of experimental groups was determined using Student’s t test or one-way ANOVA followed Tukey by post hoc test for multiple pairwise comparisons. Data were shown as means ± SEM, ****P < 0.0001.

Lithium chloride (LiCl) is considered to affect the production of IP_3_ by interfering with the inositol phosphate metabolic cycle [41,42]. In our study, pretreatment with LiCl (1 mM) for 30 minutes significantly blocked CCh-induced Ca^2+^ signaling in both monocellular (Fig 3A-3D) and multiple QGP-1 cells. Furthermore, IP_3_R blocker 2-APB (20 μM) was identified to block CCh-enhanced transmembrane current (Fig 3E-3G) and Ca^2+^ signaling in both mononuclear cell and multiple QGP-1 cells (Fig 3H-3K). Furthermore, selective IP_3_R blocker Xestospongin C [43] (XeC, 100 nM), also significantly inhibited the CCh-induced enhancement of calcium signals (Fig 3L-3O).

These data strongly indicate that CCh combined with mAChRs to induce calcium release from the ER mediated by IP_3_/IP_3_R pathway in ECs.

### 3.3 TRPV4 channel was expressed in QGP-1 cells and participated in Ca^2+^ signaling

**Fig 4:**
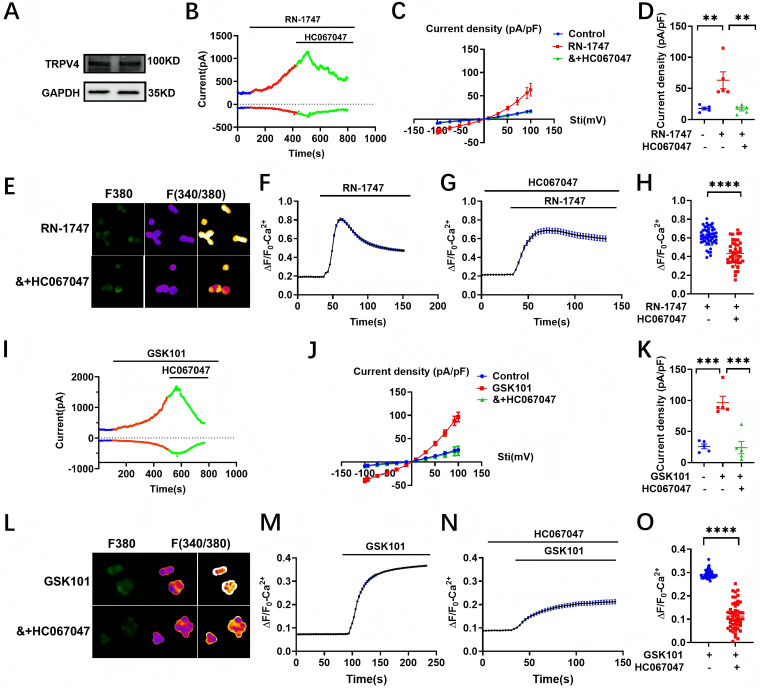
TRPV4 channel was expressed in QGP-1 cells and participated in Ca^2+^ signaling. (A) Western blot confirmed the expression of TRPV4 protein in QGP-1 cells. (n = 3). (B) RN-1747 (20 μM) can enhance transmembrane current, but this effect can be inhibited by HC067047 (10 μM). (C) Current density-voltage curves in response to voltage steps from −100 to +100 mV in the presence of RN-1747 (20 μM) or a combination of RN-1747 plus HC067047 (10 μM). (n = 5). (D) Summary data of current density measured at 100 millivolts. (n = 5). (E) Shows the changes in fluorescence ratio induced by RN-1747 (20 μM) alone and RN-1747 (20 μM) + HC067047 (10 μM). (F) Summary of time-course data for calcium signal transduction induced by RN-1747 (20 μM) in PSS solution (n = 50). (G) Summary data of the time course of calcium signals induced by RN-1747 (20 μM) in PSS solution containing 10 μM HC067047 (n = 44). (H) Comparison of calcium signals between RN-1747 and RN-1747 + HC067047 groups. (I) GSK101 (200 nM) can enhance transmembrane current, but this effect can be inhibited by HC067047 (10 μM). (J) Current density-voltage curves in response to voltage steps from −100 to +100 mV in the presence of GSK101 (100 nM) or a combination of GSK101 plus HC067047 (10 μM). (n = 5). (K) Summary data of current density measured at 100 mV. (n = 5). (L) Shows the changes in fluorescence ratio induced by GSK101 (200 nM) alone and GSK101 (200 nM) + HC067047 (10 μM). (M) Summary of time-course data for calcium signal transduction induced by GSK101 (200 nM) in PSS solution (n = 51). (N) Summary data of the time course of calcium signals induced by GSK101 (200 nM) in PSS solution containing 10 μM HC067047 (n = 56). (O) Comparison of calcium signals between GSK101 and GSK101 + HC067047 groups. The statistical significance of differences in the means of experimental groups was determined using Student’s t test or one-way ANOVA followed Tukey by post hoc test for multiple pairwise comparisons. Data were shown as means ± SEM, **P < 0.01, ***P < 0.001, ****P < 0.0001.

TRPV4 channel is a non-selective cation channel with high permeability to Ca^2+^. TRPV4 could been activated by IP_3_R/[Ca^2+^]_i_ signaling pathway in various functional cells to particicate in multiple physiological and pathological processes [39,44]. At present, the expression and function of TRPV4 channels in QGP-1 cells have not been reported yet. Our study aimed to test the role of TRPV4 in QGP-1 cells. First, Western blot analysis confirmed that TRPV4 was indeed expressed in QGP‑1 cells (Fig 4A). As shown in Fig 4B and 4I, the selective TRPV4 activator RN‑1747 (20 μM) and another selective agonist GSK1016790A (GSK101, 200 nM) both enhanced the membrane non‑selective cation current and increased the non‑selective cation current density. These effects were reversed by the selective TRPV4 blocker HC067047 (10 μM) (Fig 4C, 4D, 4J, 4K). Statistical analysis of multiple cells further confirmed that HC067047 significantly inhibited the current density and Ca^2+^ signals enhanced by RN‑1747 and GSK101 (Fig 4E–4H, 4L–4O).

Collectively, these results provide strong evidence for the expression and function of TRPV4 channels in QGP‑1 cells.

### 3.4 TRPV4 channel was involved in cholinergic signaling in QGP-1 cells

**Fig 5:**
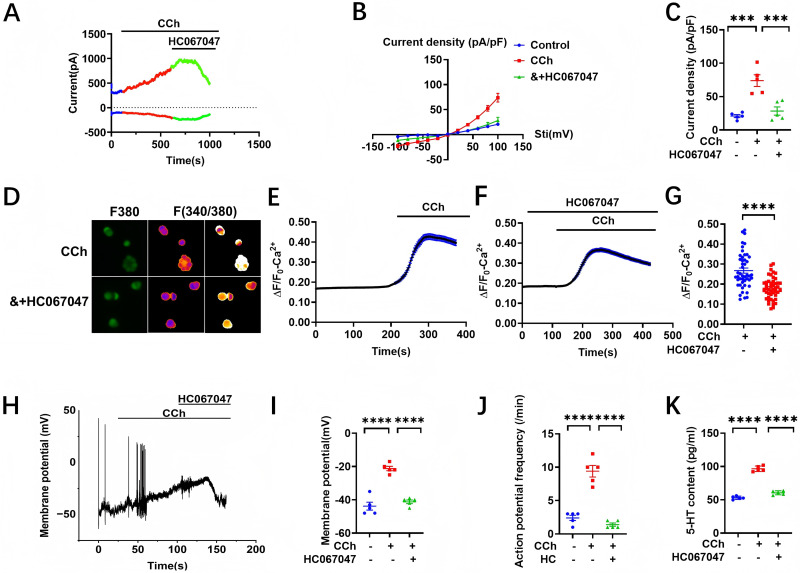
TRPV4 channel was involved in cholinergic signaling in QGP-1 cells. (A) CCh (20 μM) increases transmembrane current, and this effect is inhibited by HC067047 (10 μM). (B) Current density-voltage curves in response to voltage steps from −100 to +100 mV in the presence of CCh (20 μM) or a combination of CCh plus HC067047 (10 μM). (n = 5). (C) Summary data of current density measured at 100 millivolts. (n = 5). (D) Shows the changes in fluorescence ratio induced by CCh (20 μM) alone and CCh (20 μM) + HC067047 (10 μM). (E) Summary of time-course data for calcium signal transduction induced by CCh (20 μM) in PSS solution (n = 47). (F) Summary data of the time course of calcium signals induced by CCh (20 μM) in PSS solution containing 10 μM HC067047 (n = 46). (G) Comparison of calcium signals between CCh and CCh + HC067047 groups. (H) Time-voltage graph showing the significant changes in QGP-1 membrane potential and action potential frequency induced by CCh at a clamped current of 0 pA under the action of HC067047 (10 μM). (I) Comparison graph of CCh-induced action potential frequency versus the CCh + HC067047 group at a clamped current of 0 pA under the action of HC067047 (10 μM). (J) Comparison graph of CCh-induced membrane potential change amplitude versus the CCh + HC067047 group at a clamped current of 0 pA under the action of HC067047 (10 μM). (K) Comparison of changes in 5-HT content in cell culture medium under the treatment conditions of CCh (20 μM) and CCh (20 μM) + HC067047 (10 μM). The statistical significance of differences in the means of experimental groups was determined using Student’s t test or one-way ANOVA followed Tukey by post hoc test for multiple pairwise comparisons. Data were shown as means ± SEM, ***P < 0.001, ****P < 0.0001.

After confirming the expression and function of TRPV4 in QGP-1 cells, we further investigated whether the TRPV4 channel is located downstream of the IP_3_/IP_3_R pathway to participate in cholinergic signaling and whether it is involved in 5-HT secretion. As shown in Fig 5A, the membrane current and non-selective cation current density of QGP-1 cells induced by CCh (20 μM) were obviously abolished by HC067047(10 μM). Statistical analysis of multiple cells further confirmed the effect of HC067047 (Fig 5B, 5C). We also found that HC067047 significantly suppressed CCh-induced Ca^2+^ signaling in QGP-1 cells (Figs 5D-5G). These results strongly indicated that TRPV4 channels are physiologically involved in cholinergic signaling in QGP-1 cells. Sequently, we verified the function of TRPV4 by measuring action potentials. As shown in Fig 5H, under the condition of a holding current of 0 pA, increased membrane potential and action potential frequency were induced by CCh. Noticeably, TRPV4 inhibitor HC067047 significantly reversed CCh’ effect by reducing membrane potential and action potential frequency in multiple QGP-1 cells (Figs 5I, 5J). Which further illustrates that TRPV4 plays a key role in the function of 5-HT secretion in QGP-1 cells.

Furthermore, ELISA assay was performed to detect the secretion of 5-HT in QGP-1 cells in the occurrence of TRPV4 inhibitor HC067047(10 μM). As expected, ACh-dependent secretion level of 5-HT was significantly decreased with the treatment of HC067047 (Fig 5K).

Above study demonstrated that TRPV4 plays a key role in the function of 5-HT secretion in QGP-1 cells.

### 3.5 TRPV4 as a candidate molecule for SOCE regulates CCh-induced Ca^2+^ signaling

**Fig 6:**
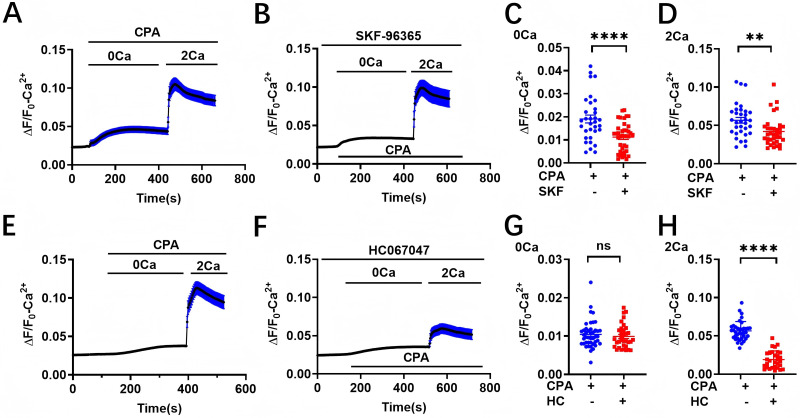
CPA-induced SOCE in QGP-1 cells. (A) Time course of changes in the fluorescence ratio [Ca^2+^]_i_ induced by 10 μM CPA under 0 Ca (calcium-free) conditions, followed by the addition of 2 mM Ca (2 Ca, Ca^2+^ add-back). (n = 34). (B) Data show the effect of SKF-96365 (50 μM) on the time course of CPA-induced calcium signaling under both extracellular 0 Ca and extracellular 2 Ca conditions. (n = 40). (C) Comparison of calcium signals between the CPA group and CPA + SKF-96365 group under 0 Ca conditions. (D) Comparison of calcium signals between the CPA group and the CPA + SKF-96365 group under 2 Ca conditions. (E) Time course of changes in the fluorescence ratio [Ca^2+^]_i_ induced by 10 μM CPA under 0 Ca conditions, followed by the addition of 2 Ca. (n = 43). (F) Data show the effect of HC067047 (10 μM) on the time course of CPA-induced calcium signaling under both extracellular 0 Ca and extracellular 2 Ca conditions. (n = 34). (G) Comparison of calcium signals between the CPA group and CPA + HC067047 group under 0 Ca conditions. (D) Comparison of calcium signals between the CPA group and the CPA + HC067047 group under 2 Ca conditions. The statistical significance of differences in the means of experimental groups was determined using Student’s t test or one-way ANOVA followed Tukey by post hoc test for multiple pairwise comparisons. Data were shown as means ± SEM, **P < 0.01, ****P < 0.0001.

**Fig 7:**
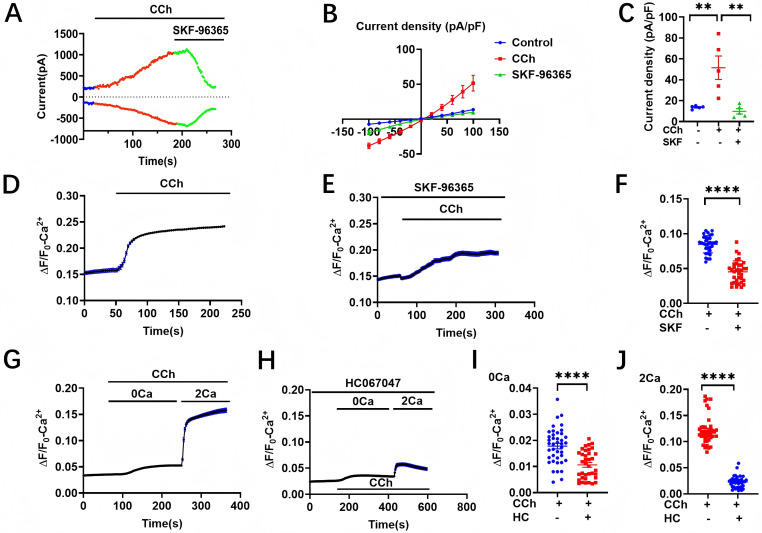
TRPV4 regulates CCh-induced Ca^2+^ signaling via the SOCE. (A) CCh (20 μM) increases transmembrane current, and this effect is inhibited by SKF-96365 (50 μM). (B) Current density-voltage curves in response to voltage steps from −100 to +100 mV in the presence of CCh (20 μM) or a combination of CCh plus SKF-96365 (50 μM). (n = 5). (C) Summary data of current density measured at 100 millivolts. (n = 5). (D) Summary of time-course data for calcium signal transduction induced by CCh (20 μM) in PSS solution (n = 33). (E) Summary data of the time course of calcium signals induced by CCh (20 μM) in PSS solution containing 50 μM SKF-96365 (n = 35). (F) Comparison of calcium signals between CCh and CCh + SKF-96365 groups. (G) Time course of changes in the fluorescence ratio [Ca^2+^]i induced by 20 μM CCh under 0 Ca (calcium-free) conditions, followed by the addition of 2 mM Ca (2 Ca, Ca^2+^ add-back). (n = 41). (H) Data show the effect of HC067047 (10 μM) on the time course of CCh-induced calcium signaling under both extracellular 0 Ca and extracellular 2 Ca conditions. (n = 36). (I) Comparison of calcium signals between the CCh group and CCh + HC067047 group under 0 Ca conditions. (J) Comparison of calcium signals between the CCh group and the CCh + HC067047 group under 2 Ca conditions. The statistical significance of differences in the means of experimental groups was determined using Student’s t test or one-way ANOVA followed Tukey by post hoc test for multiple pairwise comparisons. Data were shown as means ± SEM, **P < 0.01, ****P < 0.0001.

It is well known that [Ca^2+^]_i_ release from the endoplasmic reticulum (ER) triggers store-operated calcium entry (SOCE) [45], an essential physiological process in cells. We investigated whether the SOCE mechanism is involved in CCh-induced Ca^2+^ signaling. As shown in Fig 6A–6D, functional SOCE was confirmed in QGP‑1 cells. Cyclopiazonic acid (CPA, 10 μM), an inhibitor of ER Ca^2+^-ATPase, induced SOCE by depleting ER [Ca^2+^]_i_ stores, and this effect was significantly inhibited by the selective SOCE blocker SKF96365 (SKF, 50 μM). Previous studies have shown that some TRP channels are molecular candidates for SOCE. Consistent with this, our study found that HC067047 (10 μM) partially inhibited CPA-evoked SOCE in QGP‑1 cells (Fig 6E–6H). These results suggest that TRPV4 channels may be molecular components of SOCE. To further verify whether TRPV4-mediated SOCE is involved in CCh-induced Ca^2+^ signaling, cells were treated with the SOCE inhibitor SKF96365, which significantly reduced the CCh-induced enhancement of current density and the elevation of Ca^2+^ signals (Fig 7A–7F). In addition, HC067047 (10 μM) significantly inhibited CCh-induced Ca^2+^ signaling in 2Ca PSS solution (Fig 7G–7J).

These results indicate that TRPV4-constituted SOCE is critical for CCh-induced Ca^2+^ signaling in QGP‑1 cells.

### 3.6 TRPV4-mediated calcium influx activated RyR, and enhanced calcium signaling through CICR mechanism

**Fig 8:**
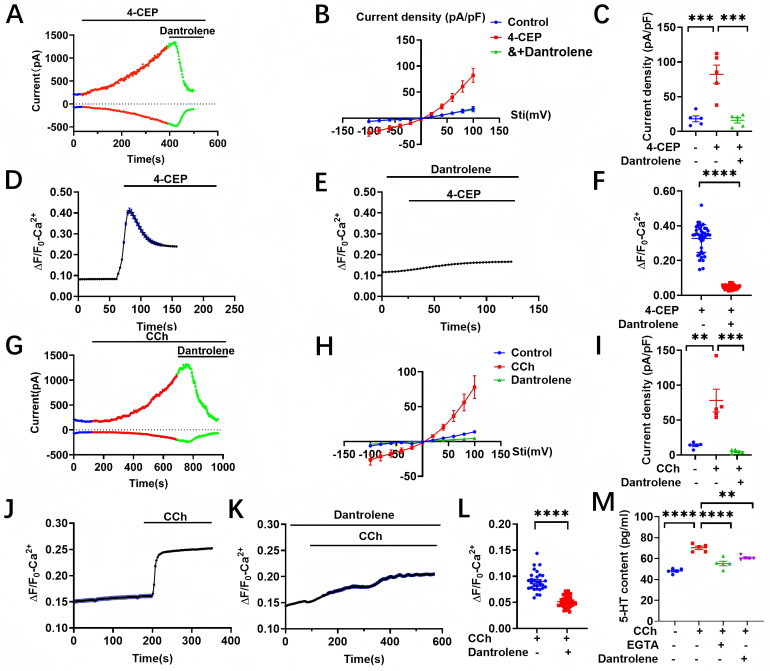
TRPV4-mediated calcium influx activated RyR, and enhanced calcium signaling through CICR mechanism. (A) 4-CEP (1 μM) increases transmembrane current, and this effect is inhibited by Dantrolene (100 μM). (B)Current density-voltage curves in response to voltage steps from −100 to +100 mV in the presence of 4-CEP (1 μM) or a combination of 4-CEP (1 μM) plus Dantrolene (100 μM). (n = 5). (C) Summary data of current density measured at 100 millivolts. (n = 5). (D) Summary of time-course data for calcium signal transduction induced by 4-CEP (1 μM) in PSS solution (n = 40). (E) Summary data of the time course of calcium signals induced by 4-CEP (1 μM) in PSS solution containing 100 μM Dantrolene (n = 39). (F) Comparison of calcium signals between 4-CEP and 4-CEP + Dantrolene groups. (G) CCh (20 μM) increases transmembrane current, and this effect is inhibited by Dantrolene (100 μM). (H) Current density-voltage curves in response to voltage steps from −100 to +100 mV in the presence of CCh(20 μM) or a combination of CCh(20 μM) plus Dantrolene (100 μM). (n = 5). (I) Summary data of current density measured at 100 millivolts. (n = 5). (J) Summary of time-course data for calcium signal transduction induced by CCh (20 μM) in PSS solution (n = 52). (K) Summary data of the time course of calcium signals induced by CCh (20 μM) in PSS solution containing 100 μM Dantrolene (n = 44). (L) Comparison of calcium signals between CCh and CCh + Dantrolene groups. (M) Comparison of changes in 5-HT levels in cell culture medium among the CCh (20 μM) single treatment group, CCh (20 μM) + EGTA (0.5 mM) co-treatment group, and CCh (20 μM) + Dantrolene (100 μM) co-treatment group. The statistical significance of differences in the means of experimental groups was determined using Student’s t test or one-way ANOVA followed Tukey by post hoc test for multiple pairwise comparisons. Data were shown as means ± SEM, **P < 0.01, ***P < 0.001, ****P < 0.0001.

It is known that in excitable cells, the entry of extracellular calcium can indirectly activate ryanodine receptors (RyRs), leading to the massive release of calcium ions from the sarcoplasmic reticulum or endoplasmic reticulum into the cytoplasm [46,47]. This process is called Calcium-Induced Calcium Release (CICR). This mechanism is involved in the secretion of various hormones in vivo [48]. Therefore, this study aims to investigate whether CICR is involved in the transmission of cholinergic signaling in QGP-1 cells. First, the RyR-selective agonist 4-CEP (1 μM) enhanced the membrane current and non-selective cation current density in QGP-1 cells (Figs 8A, 8B), and these effects were abolished by the RyR-selective blocker Dantrolene (100 μM). Statistical analysis of multiple cells further confirmed that dantrolene (100 μM) significantly reversed the 4-CEP (1 μM)-induced increases in current density (Fig 8C) and Ca^2+^ signals (Fig 8D–8F).

To further determine whether RyRs are involved in cholinergic signaling, CCh (20 μM) was applied to QGP-1 cells instead of 4-CEP (1 μM). As shown in Fig 8G–8L, the CCh-induced increases in current density and intracellular Ca^2+^ signals at both the single-cell and multi-cell levels were inhibited by Dantrolene. These results strongly indicate that in QGP-1 cells, RyRs are physiologically involved in cholinergic signaling. After ACh/TRPV4-induced extracellular calcium influx, RyRs were activated, triggering the CICR mechanism and thereby further amplifying the calcium signal.

Based on the above cellular experiments, we systematically assessed the specific effects of various calcium signaling pathways on 5-HT secretion by combining specific antagonists with ELISA detection. First, under Ca^2+^-free conditions (EGTA, 0.5 mM), 5-HT secretion decreased markedly and was comparable to the control group, indicating that IP_3_/IP_3_R-mediated intracellular calcium release is required to maintain basal 5-HT secretion. Second, treatment with the RyR antagonist Dantrolene (100 μM) slightly reduced 5-HT levels compared with the CCh-treated group, suggesting that the CICR mechanism can partially boost 5-HT secretion (Fig 8M). Taken together, basal Ca^2+^ levels derived from intracellular calcium release are a prerequisite for physiological 5-HT secretion. Under pathological conditions, excessive activation of TRPV4 channels enhances extracellular calcium influx, which further amplifies calcium signals via the CICR mechanism and ultimately leads to excessive 5-HT secretion, thereby potentially aggravating UC symptoms.

### 3.7 The role of TRPV4 channels in the pathogenesis of ulcerative colitis

**Fig 9:**
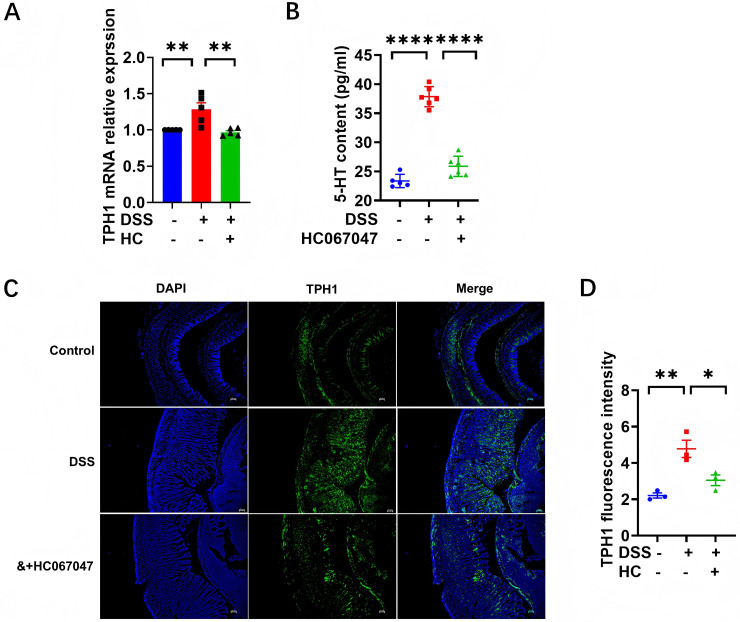
TRPV4 blockers reduce the expression of TPH1 and the secretion of 5-HT in mice with UC. (A) Summary data of TPH1 transcription in the colon of mice from the control group, DSS group, and HC067047 (10 mg·kg^-1^)-treated group(n = 5). (B) Summary data of 5-HT content in the serum of mice from the control group, DSS group, and HC067047 (10 mg·kg^-1^)-treated group (n = 5). (C) Summary data of immunofluorescence staining for TPH1 in the colon of mice from the control group, dextran sulfate sodium (DSS)-treated group, and DSS + HC067047 (10 mg·kg^-1^)-treated group (n = 3). (D) The summary graph shows the differences in TPH1 fluorescence intensity in the colon of mice from the control group (n = 3), dextran sulfate sodium (DSS)-treated group (n = 3), and DSS + HC067047 (10 mg·kg^-1^)-treated group (n = 3). The statistical significance of differences in the means of experimental groups was determined using Student’s t test or one-way ANOVA followed Tukey by post hoc test for multiple pairwise comparisons. Data were shown as means ± SEM, *P < 0.1, **P < 0.01, ***P < 0.001, ****P < 0.0001.

**Fig 10:**
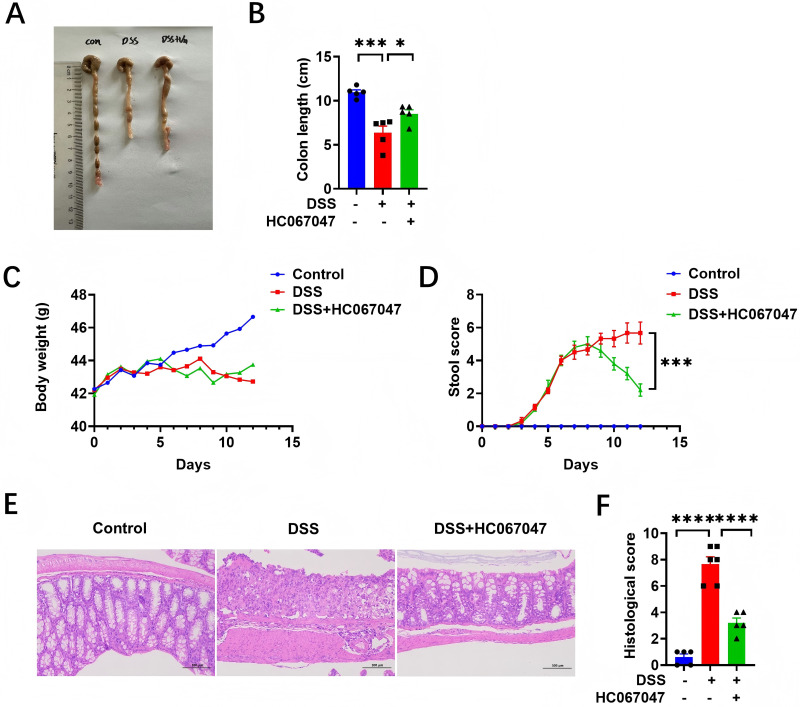
The role of TRPV4 channels in the pathogenesis of ulcerative colitis. (A and B) Summary data of colon length in mice from the control group, DSS group, and HC067047 (10 mg·kg^-1^)-treated group. (n = 5). (C) Summary data of body weight changes within 12 days in mice from the control group, DSS group, and HC067047 (10 mg·kg^-1^)-treated group. (D) Summary data of fecal score changes within 12 days in mice from the control group, DSS group, and HC067047 (10 mg·kg^-1^)-treated group. (E) The photo showing a representative Hematoxylin-and-Eosin stain of intestinal mucosa from healthy (Ctrl), DSS-treated, and DSS + HC067047 (10 mg·kg^-1^)-treated mice (magnification: × 20). (F) Summary bar graph showing the difference in histological scores of mice from the control group (n = 5), DSS group (n = 6), and DSS + HC067047 (10 mg·kg^-1^)-treated group (n = 5). The statistical significance of differences in the means of experimental groups was determined using Student’s t test or one-way ANOVA followed Tukey by post hoc test for multiple pairwise comparisons. Data were shown as means ± SEM, *P < 0.1, ***P < 0.001, ****P < 0.0001.

In vitro experiments demonstrated that the TRPV4 channel acts as a key regulatory node underlying acetylcholine-induced excessive 5-HT secretion. In our in vivo study, we aim to verify whether TRPV4 aggravates the clinical manifestations of UC by triggering overproduction of 5-HT. The qPCR data indicated that the transcriptional level of TPH1 in the colon of DSS-induced mice was significantly increased, and supressed by HC067047 (10 mg·kg^-1^) treatment (Fig 9A). Consistently, serum 5-HT secretion was increased in the mice from DSS group and decreased in the mice with HC 067047 (10 mg·kg^-1^) treatment (Fig 9B). Subsequently, immunofluorescence staining data showed that TPH1 expression was increased in UC mice, and decreased after treatment with the TRPV4 inhibitor (Fig 9C, 9D). Furthermore, compared with mice in the control group, the colon length of mice in the dextran sulfate sodium (DSS)-treated group was significantly shortened; whereas after treatment with HC067047 (10 mg·kg^-1^), a TRPV4 channel blocker, the colon length of mice was significantly restored (Fig 10A, 10B). We monitored changes in mouse body weight and fecal score. As shown in Fig 10C and 10D, after treatment with the TRPV4 inhibitor, the body weight and fecal index of mice were improved compared with those in the DSS group. Histological examination of the mouse colon showed that compared with the control group, the DSS-treated mice’ colon had obvious inflammatory cell infiltration and significant damage on the colonic mucosa. These symptoms were gradually alleviated after treatment with the TRPV4 inhibitor (Fig 10E, 10F). Above data indicated that TRPV4 channels play a key role in the synthesis and secretion of 5-HT and are involved in the occurrence and development of UC.

## 4 Discussion

**Fig 11:**
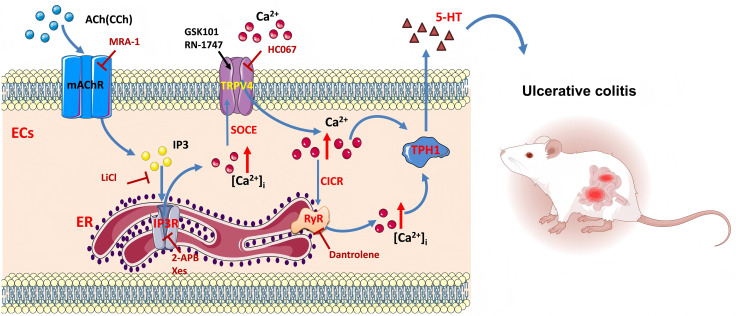
Schematic diagram of the mechanism by whi ch vagus nerve excitation participates in the pathogenesi s of UC via the mAChR/IP_3_/IP_3_R/SOCE/TRPV4/CICR/Ca^2+^/5‑HT pathway. Vagus nerve stimulation induces the release of the neurotransmitter ACh. Upon binding to mAChR, ACh triggers IP_3_‑mediated activation of IP_3_R, thereby inducing Ca^2+^ release from the endoplasmic reticulum calcium store. Depletion of the calcium store subsequently activates SOCE, which promotes the opening of TRPV4 channels on the cell membrane and mediates extracellular Ca^2+^ influx. The influxed Ca^2+^ directly promotes 5‑HT release on one hand; on the other hand, it further amplifies intracellular Ca^2+^ signals via CICR, forming a positive feedback loop. This ultimately enhances 5‑HT release and contributes to the pathogenesis of UC.

In this study, we demonstrated for the first time that TRPV4 was involved in the regulation of calcium signaling in QGP-1 in response to vagal neurotransmitter. Furthermore, TRPV4-induced 5-HT release in QGP-1 participated in the progression of UC. This conclusion is supported by the following evidence: (1) TRPV4 was expressed in QGP-1 and regulated changes in intracellular calcium signaling; (2) TRPV4 was essential for ACh-induced calcium-dependent 5-HT release from QGP-1; and (3) TRPV4-mediated changes in calcium signaling exacerbated UC symptoms by promoting 5-HT release. Therefore, we believe that this study has deepened our understanding of the novel molecular mechanism underlying vagal neurotransmitter-induced 5-HT release from QGP‑1 cells, and suggests that TRPV4 may serve as a potential therapeutic target for UC (Fig 11).

Previous studies have demonstrated that the vagal neurotransmitter ACh can regulate the release of 5-HT by modulating Ca^2+^ signaling in ECs [6]. Intracellular calcium concentration was crucial for the release of 5-HT, but the specific source of calcium ions remained unclear. Generally, cellular stimulation elevated intracellular calcium concentration via two distinct pathways: one involved the release of stored intracellular calcium ions ([Ca^2+^]_i_) from the endoplasmic reticulum/sarcoplasmic reticulum (ER/SR) [49], and the other referred to the influx of extracellular Ca^2+^ through calcium-permeable cation channels. In this study, we found that selective blockade of mAChRs attenuated the enhancement of calcium signals triggered by CCh, an acetylcholine analog. Additionally, interfering with the synthesis of inositol IP_3_ and specifically blocking inositol IP_3_Rs could suppress the CCh-induced Ca^2+^ signaling and membrane currents. Above evidence indicated that mAChR-dependent cholinergic signals activated the IP_3_/IP_3_R pathway, thereby triggering the release of ER-stored [Ca^2+^]i in QGP-1 cells. When cells were further stimulated with CCh under Ca^2+^‑free extracellular conditions, 5-HT secretion decreased significantly relative to the CCh group and only remained at the basal level. This suggests that calcium released from endoplasmic reticulum calcium stores primarily maintains basal secretion, while robust and sustained CCh-evoked 5-HT secretion depends on extracellular calcium influx.

A reduction in endoplasmic reticulum [Ca^2+^]_i_ concentration serves as a key signal triggering SOCE, which in turn activates a series of Ca^2+^-permeable cation channels such as TRP channels [39,44,50]. Among these, the TRPV4 channel has attracted increasing attention due to its Ca^2+^ sensitivity and pharmacological activity in UC [29,51,52]. The present study confirmed that TRPV4 channels in QGP‑1 cells, as a candidate molecule of SOCE, can be activated by intracellular Ca^2+^ signals (such as CCh‑induced elevation of [Ca^2+^]_i_), thereby mediating the regulation of 5‑HT secretion. This conclusion is supported by the following evidence: 1) TRPV4 is expressed and functionally active in QGP‑1 cells; 2) TRPV4 channel inhibitors suppress CCh‑induced increases in Ca^2+^ signals, transmembrane currents, and action potential frequency; 3) Selective blockade of TRPV4 channels reduces 5‑HT secretion in QGP‑1 cells; 4) TRPV4 channels participate in CCh‑induced Ca^2+^ signaling by regulating SOCE‑mediated extracellular Ca^2+^ influx. Based on the above results, we propose that TRPV4, a key component of SOCE, can be activated by CCh-induced elevation of intracellular calcium, thereby regulating 5-HT secretion. Collectively, intracellular calcium release from the endoplasmic reticulum exerts dual functions: it not only sustains basal cellular 5-HT secretion, but also triggers SOCE by depleting endoplasmic reticulum calcium stores, which provides an essential initiating signal for TRPV4 channel activation and mediates cholinergic signal-induced functional 5-HT secretion.

CICR was a core positive feedback mechanism for amplifying calcium signals in excitable cells, and its key lay in the sensitivity of RyR to cytoplasmic calcium concentration [53,54]. As a model of ECs, QGP-1 cells were also classified as excitable cells. This study further investigated whether cholinergic signals could further amplify calcium signals through the CICR mechanism. The results showed that selective blockade of RyR suppressed CCh‑induced Ca^2+^ signals and membrane currents, while moderately attenuating 5-HT secretion levels. Collectively, these data suggest that TRPV4-mediated calcium influx serves as “trigger calcium” to activate RyR, triggering secondary [Ca^2+^]_i_ elevation from endoplasmic reticulum calcium stores and forming a positive feedback loop. This cascade amplifies global calcium signals and further facilitates 5-HT release. The positive feedback loop of CICR efficiently augments the basal [Ca^2+^]_i_ signal mediated by the upstream IP_3_ pathway as well as TRPV4-dependent calcium influx, progressively intensifying calcium response magnitude, and ultimately driving robust cholinergic signal-stimulated 5-HT secretion.

These mechanistic studies suggest that TRPV4 may play a key role in 5‑HT secretion, but whether it is involved in the pathogenesis of UC remains unclear. To address this issue, we established a mouse model of UC and treated them with a TRPV4 inhibitor. The results showed that UC model mice exhibited typical symptoms including significantly shortened colon length, marked body weight loss, and a substantially increased fecal score. Histological observation revealed obvious inflammatory cell infiltration and severe mucosal damage in the colon. These pathological changes were markedly ameliorated after treatment with the TRPV4 inhibitor. In addition, compared with the model group, mice in the inhibitor‑treated group showed decreased expression of TPH1 in colonic tissue and a significant reduction in 5‑HT levels in vivo. In conclusion, TRPV4 inhibitors can effectively alleviate the symptoms of ulcerative colitis by reducing 5‑HT secretion.

This study is the first to clarify the role of TRPV4 channels in cholinergic signal-evoked 5-HT release from enterochromaffin cells, as well as its pathological relevance in UC. In QGP-1 cells, activation of mAChRs triggers [Ca^2+^]_i_ release from the endoplasmic reticulum via the IP_3_/IP_3_R pathway, a process that only sustains basal 5-HT secretion. Depletion of endoplasmic reticulum calcium stores subsequently triggers SOCE. As an essential component of SOCE, TRPV4 channels are activated to mediate extracellular Ca^2+^ influx. On one hand, the influxed Ca^2+^ directly facilitates 5-HT secretion; on the other hand, it acts as “trigger calcium” to drive RyR-dependent CICR, forming a positive feedback loop that amplifies global calcium signals. This cascade exacerbates intracellular calcium overload and drives excessive pathological 5-HT release. In a mouse model of ulcerative colitis, pharmacological inhibition of TRPV4 markedly alleviates colonic inflammation, accompanied by downregulated TPH1 expression and reduced 5-HT concentrations. Collectively, TRPV4 serves as a central target governing cholinergic regulation of 5-HT secretion, and TRPV4-targeted therapy may represent a novel therapeutic strategy for ulcerative colitis.

## Supporting information

S1 Fig Western blot detection of TRPV4 protein expression in three groups of samples. TRPV4 protein expression levels in three groups of samples were detected by Western blot, with GAPDH as the internal control. The results demonstrated that TRPV4 was expressed in QGP-1 cells.
(PDF)

## Abbreviations

5-HT: 5-hydroxytryptamine
α7nAChR: α7 nicotinic acetylcholine receptors
ACh: Acetylcholine
CAP: Cholinergic anti-inflammatory pathway
CCh: CarbAChol
CICR: Calcium-Induced Calcium Release
CPA: Cyclopiazonic acid
CRC: Colorectal cancer
DSS: Dextran sulfate sodium
ECs: Enterochromaffin cells
EEC: Enteroendocrine cell
ER: Endoplasmic reticulum
EGTA: Ethylene glycol tetraacetic acid
GI: Gastrointestinal
GSK10: GSK1016790A
IBD: Inflammatory bowel disease
IP3: Inositol 1,4,5-triphosphate
IP3R: Inositol 1,4,5-triphosphate receptor
LiCl: Lithium chloride
MRA-1: mAChR antagonist 1
mAChRs: Muscarinic acetylcholine receptors
nAChRs: Nicotinic acetylcholine receptors
PSS: Physiological salt solution
RyRs: Ryanodine receptors
SDS: Sodium dodecyl sulfate
SOCE: Store-operated calcium entry
SKF: SKF96365
SR: sarcoplasmic reticulum
TPH1: Tryptophan hydroxylase 1
TRP: Transient receptor potential
TRPV4: Transient receptor potential vanilloid 4
UC: Ulcerative colitis
XeC: Xestospongin C
