# Supplementary material for: Molecular regulatory mechanism of TRPV4-mediated calcium-dependent 5-HT secretion from enterochromaffin cells and its involvement in ulcerative colitis
Source: PLoS One. 2026 Aug 13;21(8):e0355169. doi: 10.1371/journal.pone.0355169 (PMC13472449; doi:10.1371/journal.pone.0355169)

TRPV4 GAPDH

Group1

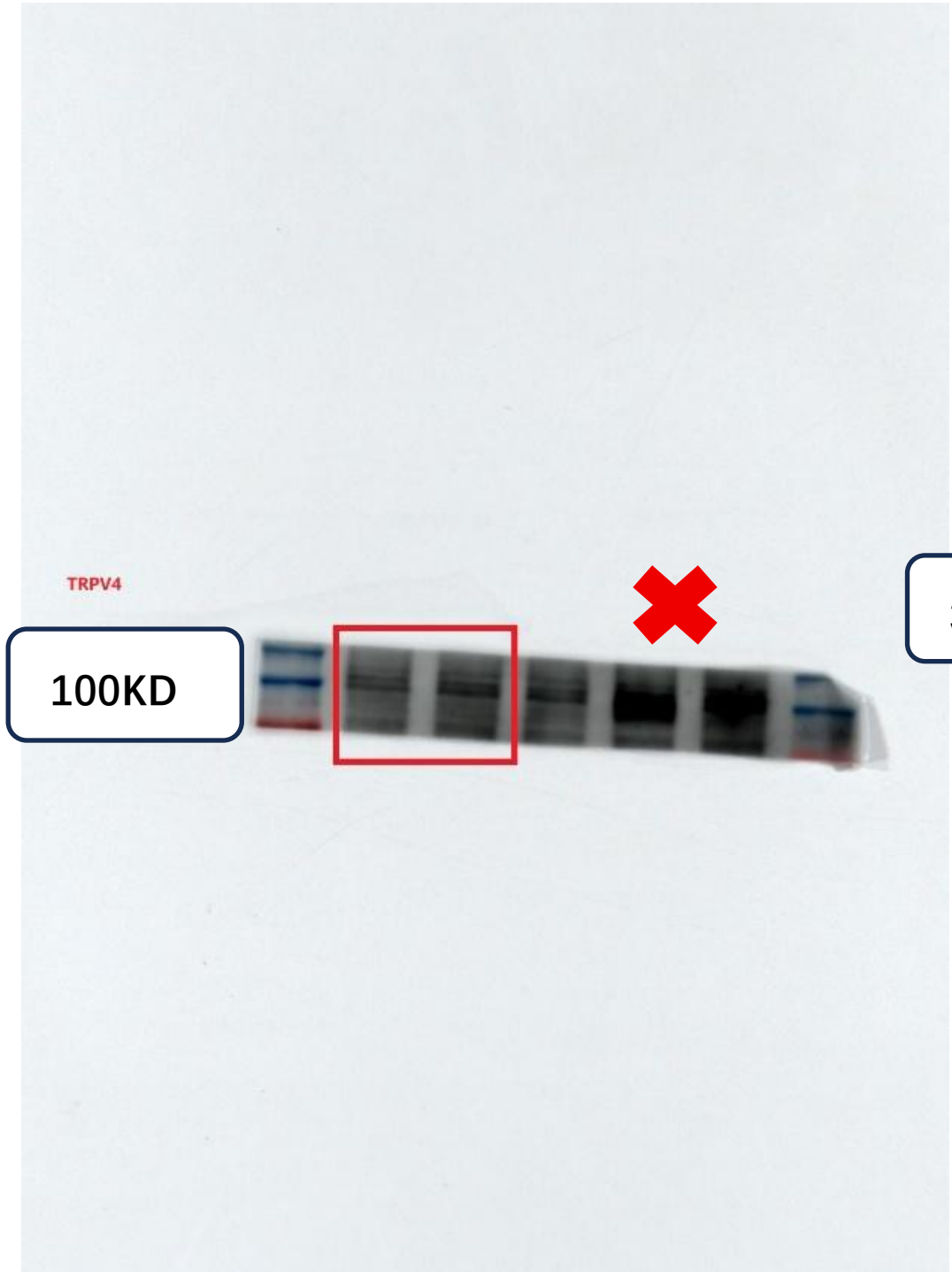

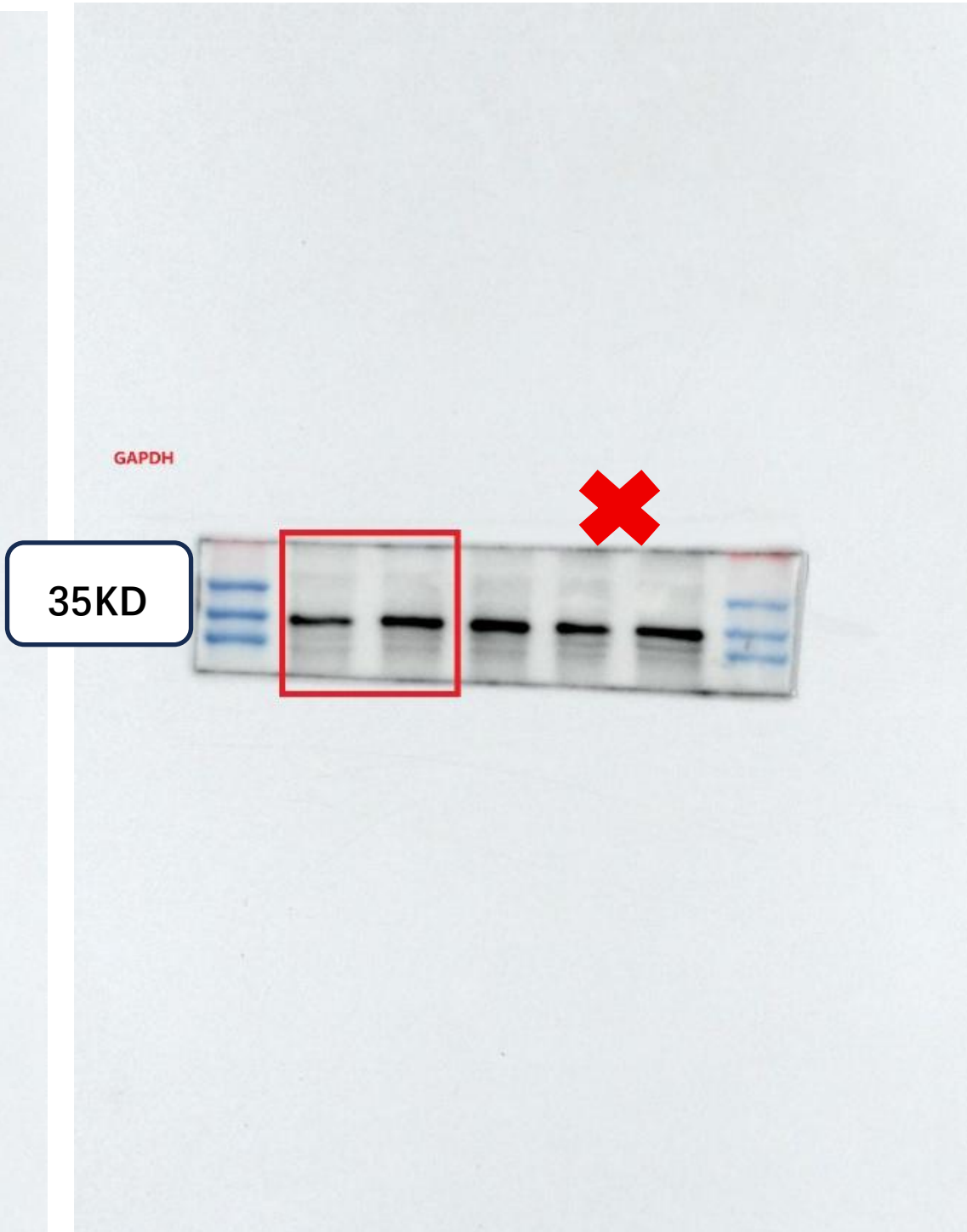

Group2（Fig 4-A）

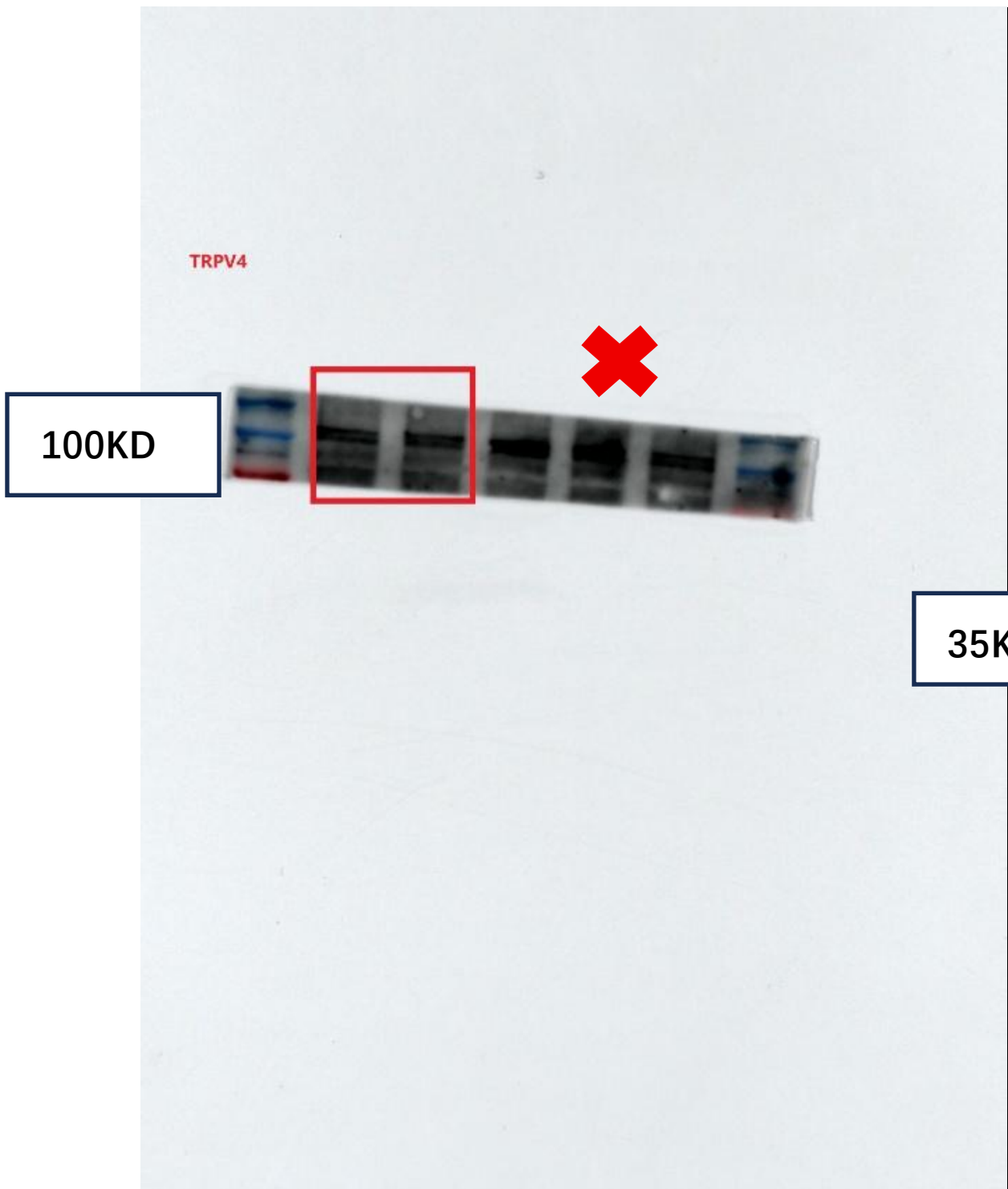

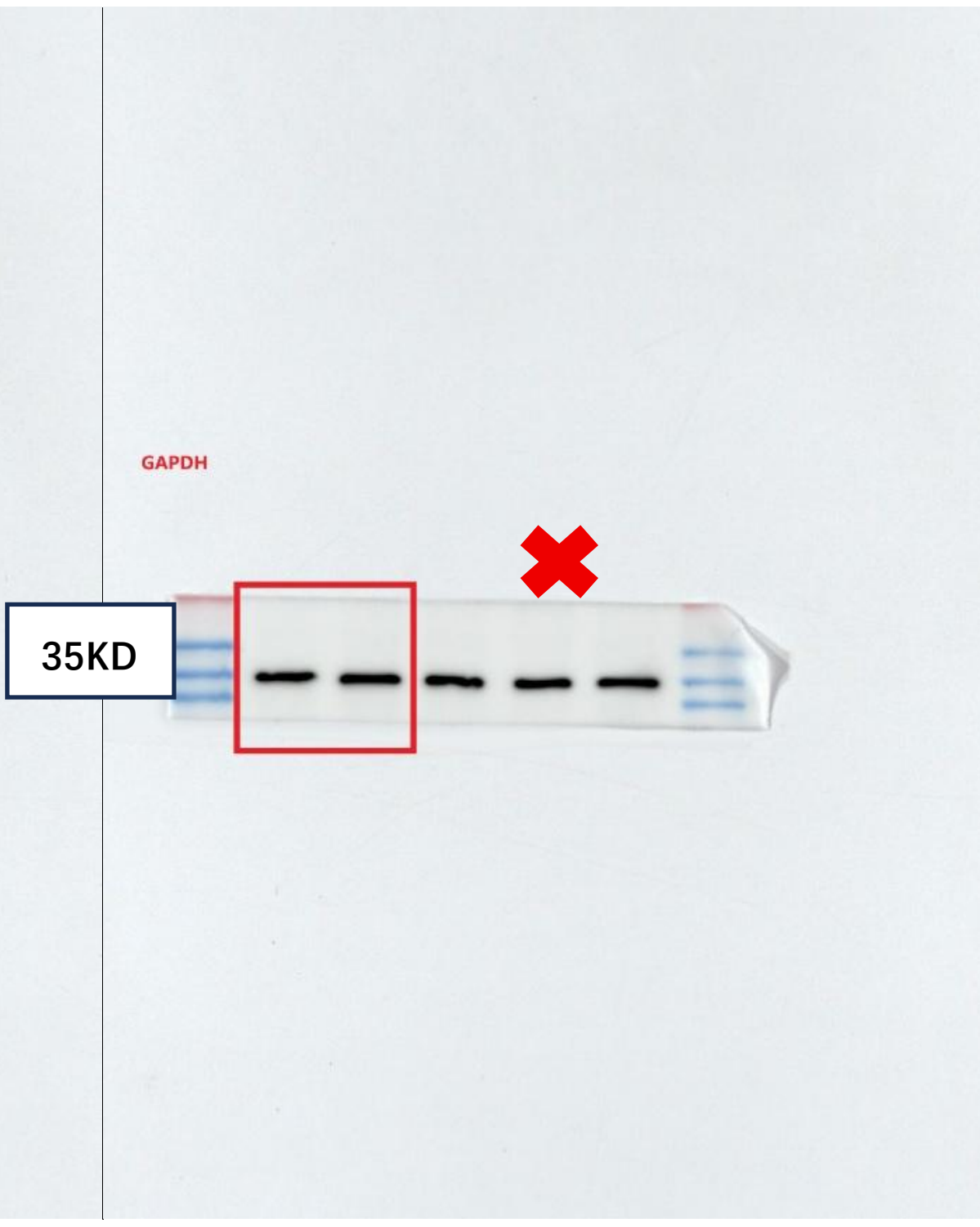

Group 3

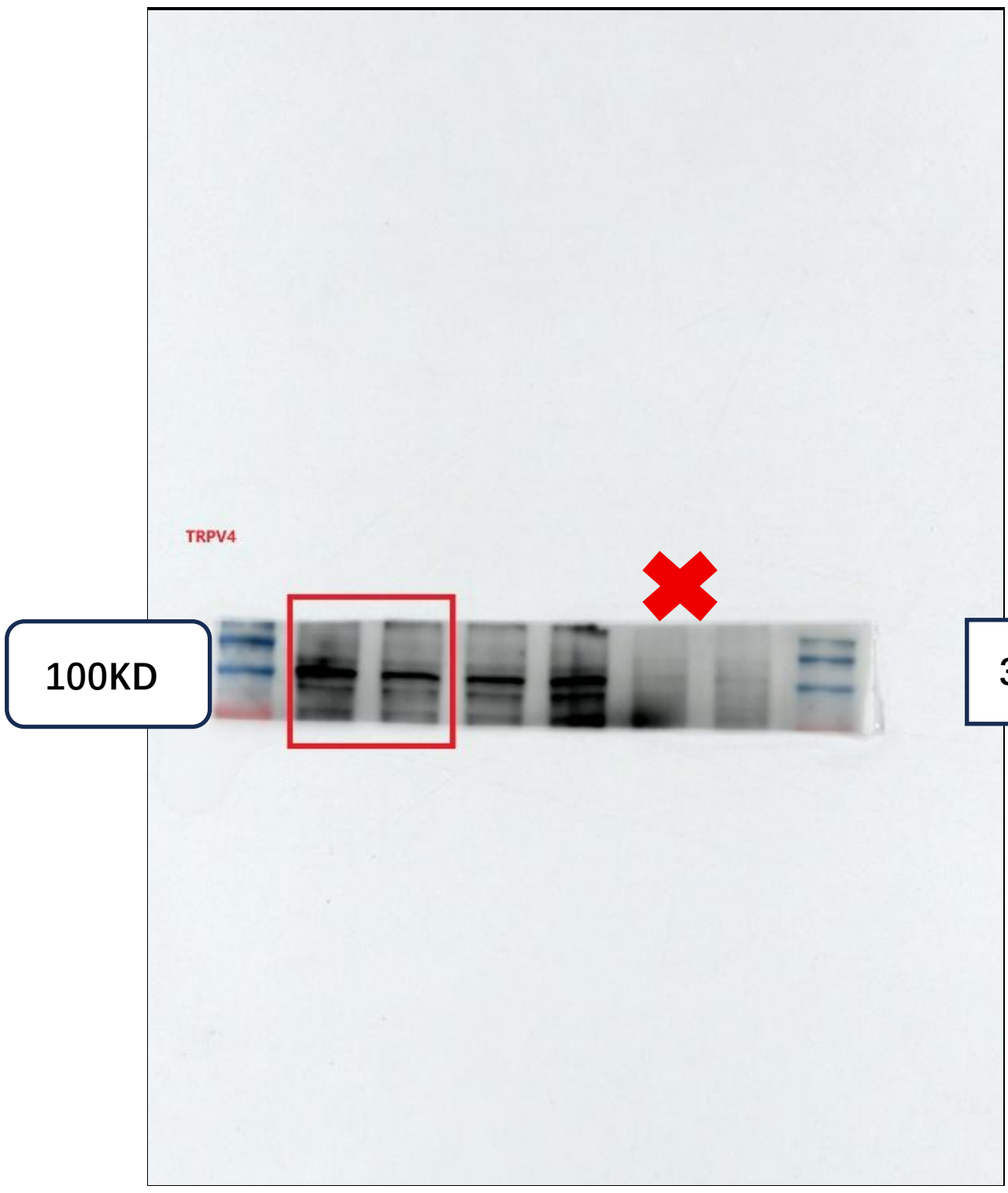

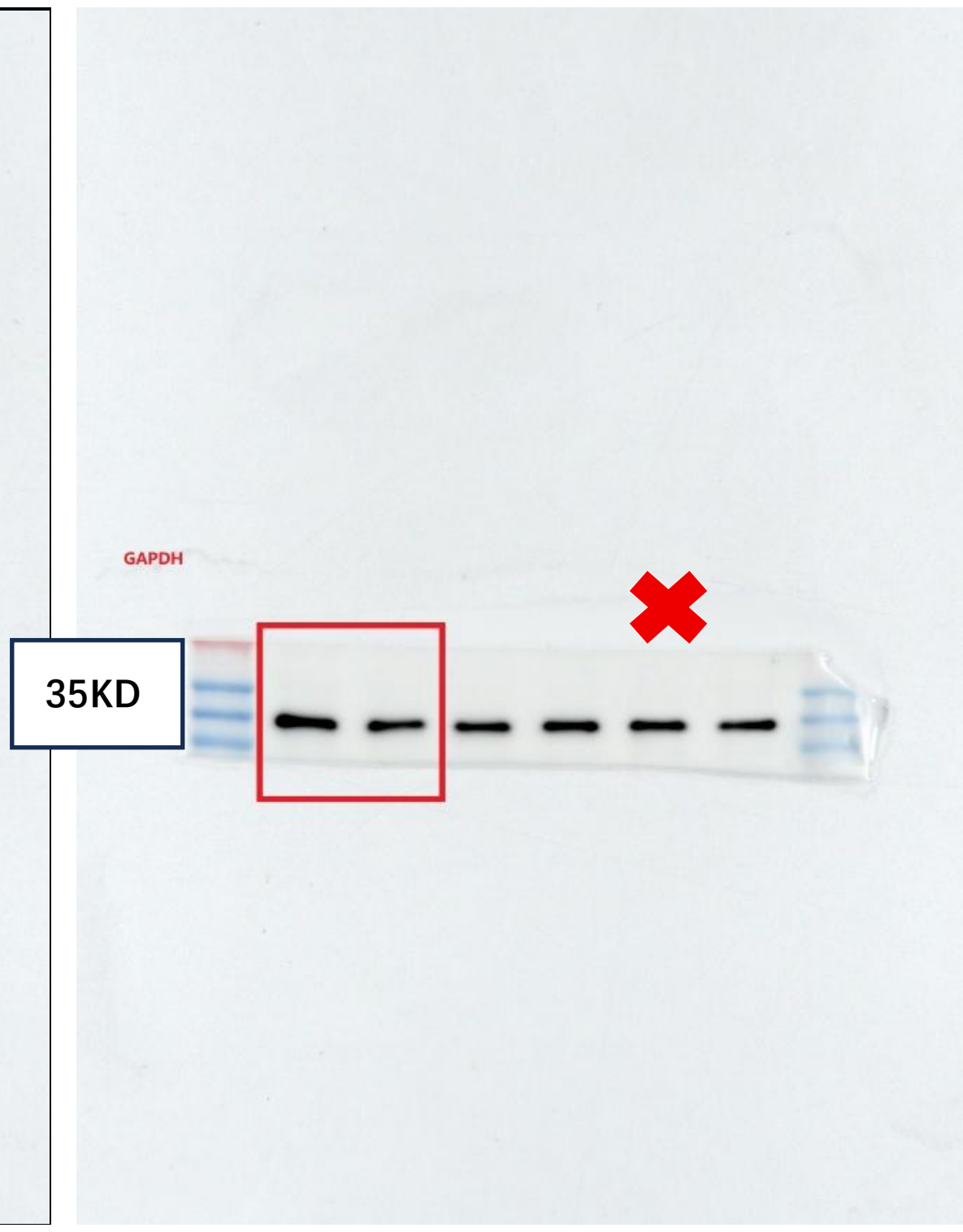

Supplement: Supplementary file 1 [file pone.0355169.s001.pdf]
